# Glucose promotes epithelial‐mesenchymal transitions in bladder cancer by regulating the functions of YAP1 and TAZ

**DOI:** 10.1111/jcmm.15653

**Published:** 2020-07-17

**Authors:** Shi Li, Hua Zhu, Hongde Chen, Jianling Xia, Fangyi Zhang, Ruoting Xu, Qi Lin

**Affiliations:** ^1^ Department of Urology The First Affiliated Hospital of Wenzhou Medical University Wenzhou China; ^2^ Department of Obstetrics and Gynecology The First Affiliated Hospital of Wenzhou Medical University Wenzhou China; ^3^ Cancer Center Sichuan Academy of Medical Sciences and Sichuan Provincial People's Hospital Hospital of the University of Electronic Science and Technology of China Chengdu China; ^4^ Department of Neurology The First Affiliated Hospital of Wenzhou Medical University Wenzhou China

**Keywords:** bladder cancer, epithelial‐mesenchymal transitions, high glucose, TAZ, YAP1

## Abstract

Glucose levels and type 2 diabetes (T2D) are both associated with tumorigenesis and epithelial‐mesenchymal transitions (EMTs). EMTs facilitate bladder cancer (BC) metastasis development, but the mechanism by which high‐glucose levels promote these EMTs in BC remains unclear. Therefore, we sought to elucidate the mechanism underlying EMT promotion due to increased glucose levels. T24 and UMUC‐3 cells were cultured in media containing different glucose concentrations. YAP1, TAZ, GLUT1 and EMT‐associated marker expression was analysed via Western blotting and qPCR. BC cell proliferation and invasion were assessed using MTT and Transwell assays, respectively. A xenograft nude mouse model of diabetes was used to evaluate tumour growth and metastasis in vivo. T2D was positively associated with pathologic grade (*P* = .016) and TNM stage (*P* < .001) in BC. High glucose triggered BC cell proliferation and invasion in both in vitro and in vivo conditions. High‐glucose levels also promoted EMTs in BC cells and increased YAP1 and TAZ expression. YAP1 or TAZ knockdown altered EMT marker expression and decreased GLUT1 expression. Overall, our results suggest that high‐glucose levels promote EMTs in BC cells via YAP1 and TAZ regulation. These effector molecules may be promising therapeutic targets for BC cases comorbid with T2D.

## 1 BACKGROUND

Bladder cancer (BC) is the fourth most common cancer among men and the second most common cause of death among urinary tract malignancies in the United States.^1^ BC is a malignant disease with high morbidity and mortality and can be divided into two categories: non–muscle‐invasive bladder cancer (NMIBC) and muscle‐invasive bladder cancer (MIBC). Nearly 75%‐80% of newly diagnosed cases of BC are NMIBC (stages Ta, T1, or Tis)^2^; however, more than half of these cases will relapse and progress to MIBC (T2 or greater) following transurethral resection of the bladder tumour (TURBT) and adjuvant chemoradiotherapy.^3^ Epithelial‐mesenchymal transitions (EMTs), the process by which epithelial cells assume a mesenchymal cell phenotype, are critical in various human cancers,^4^ including BC.^5^, ^6^ Therefore, identification of the molecular mechanisms involved in BC progression relevant EMTs is essential to the development of BC treatments.

Type 2 diabetes (T2D) has been implicated in the development of several types of cancer, including liver cancer^7^ and BC.^8^ Previous studies have demonstrated that high‐glucose levels may promote EMTs. Most of these studies focused on complications common to T2D, such as diabetic nephropathy^9^, ^10^ and macrovascular problems.^11^ Related studies with regard to cancers are limited. Additionally, the expression and stability of Yes‐associated protein 1 (YAP1) are positively correlated with O‐GlcNAcylation in high‐glucose‐stimulated liver tumorigenesis.^12^ YAP1 and TAZ (the transcriptional coactivator with PDZ‐binding motif) are key components in the conserved Hippo pathway that controls carcinogenesis, regeneration and metabolism. The explicit carcinogenicity of YAP1/TAZ has been observed in various human cancers,^13^ and YAP1/TAZ‐induced EMTs have also been reported.^14^, ^15^, ^16^ These findings suggest a potential effect of high‐glucose levels on EMT frequency via the Hippo pathway in BC cells, although the underlying mechanism remains unclear. We therefore sought to study how high‐glucose levels promote EMTs through the Hippo pathway in BC.

## 2 MATERIALS AND METHODS

### 2.1 Patients and tissue specimens

This study examined 100 BC patients, each with and without T2D, who had undergone radical cystectomy, partial cystectomy or TURBT at either the Fist Affiliated Hospital of Wenzhou Medical University or Sichuan Provincial People's Hospital from 2013 to 2018. Specimens were taken with informed consent prior to surgery and were staged according to the 2010 AJCC Cancer Staging System. All procedures were approved by the Hospital's Ethics Review Board.

### 2.2 Cell culture and transfection

Human BC cell lines (T24 and UMUC‐3) were used. Cells were maintained in RPMI‐1640 (Invitrogen), supplemented with 10% foetal bovine serum (Bio Basic) and incubated at 37°C in 5% CO_2_. Cell culture media with different glucose concentrations (2.8 and 25 mmol/L) were used in further experiments. siRNAs (No:siG000010413B and siG14212135417, RiboBio) or pc‐DNAs were transiently transfected into cells using Lipofectamine 2000 (Invitrogen). A transfection efficiency of ≥55% was deemed adequate for subsequent experiments.

### 2.3 Immunohistochemistry

Immunohistochemistry (IHC) analyses were performed using formalin‐fixed, paraffin‐embedded (FFPE) tissues. The intensity of immunostaining in tumour tissues was assessed by the subjective visual scoring of brown stains by two independent evaluators.

### 2.4 Quantitative real‐time PCR

Total RNA was isolated from tissue samples or cells using TRIzol reagent (Invitrogen), and cDNA was synthesized according to the manufacturer's recommended protocols (Thermo Scientific). Quantitative real‐time PCR (qPCR) was performed using a standard SYBR Green PCR Kit (Sangon Biotech) and an Applied Biosystems 7500 Real‐Time PCR System (Applied Biosystems). We used glyceraldehyde‐3‐phosphate dehydrogenase (GAPDH) for normalization. Primer sequences are summarized in Table S1.

### 2.5 Western blotting

Tissues and cells were lysed and analysed via Western blotting as previously described.^17^ The following primary and secondary antibodies were used: rabbit polyclonal anti‐YAP1 (1:1500, ABclonal), rabbit monoclonal anti‐pYAP1 (1:10000, Abcam), rabbit polyclonal anti‐TAZ (1:1000, LifeSpan Biosciences), mouse monoclonal anti‐GLUT1 (1:500, Santa), mouse monoclonal anti‐E‐cadherin (1:500, Santa), mouse monoclonal anti‐Vimentin (1:1000, Santa), rabbit polyclonal anti‐N‐Cadherin (1:1000, CST), rabbit polyclonal anti‐Fibronectin (1:10000, Abcam) and rabbit polyclonal anti‐GAPDH (1:1000, Sangon Biotech). Horseradish peroxidase–conjugated secondary antibodies were purchased from Cell Signaling Technology (1:2000, CST).

### 2.6 Cell viability assays (MTT)

Cell viability was assessed using an MTT Cell Proliferation and Cytotoxicity Assay Kit (Bio Basic). BC cells were seeded in 96‐well plates at a density of 5 × 10^3^ cells per well. Viability was assessed at various time points (0, 24, 48, 72 and 96 hours) by measuring the absorbance of formazan at 570 nm using a microplate reader (Thermo Scientific).

### 2.7 Transwell cell invasion assays

Cell invasion assays were conducted as follows: 1 × 10^5^ cells suspended in 200 μL of serum‐free medium were seeded on membranes precoated with 24 mg/mL Matrigel (BD Biosciences) and inserted into the Transwell apparatus (Corning Life Sciences). Medium containing 20% foetal bovine serum was used as a chemoattractant in the lower chamber. Cells were incubated at 37°C for 24 hours to allow for invasion. Cells on the lower surface were fixed in 4% paraformaldehyde and stained with Wright Giemsa (Sangon). In each replicate, cells were counted under a microscope (Olympus; ×200) in six predetermined fields. Assays were independently repeated at least three times.

### 2.8 Enzyme‐linked immunosorbent assay

The LKB1 activities and lactate concentration in the culture medium were detected using enzyme‐linked immunosorbent assay (ELISA) kits (Laibio) according to the manufacturer protocol. A microplate reader (Thermo Scientific) was used to read the absorbance of each well at 450 nm.

### 2.9 Xenograft model

To evaluate whether high‐glucose levels would affect BC tumour growth and metastasis in vivo, we established a streptozotocin (STZ)‐induced xenograft nude mouse model of diabetes.^18^ Mice were randomly divided into two groups (7 mice per group): The control group was fed a normal diet and injected with insulin to maintain blood glucose at levels lower than 16.7 mmol/L and was considered euglycemic; the treatment group was fed a high‐glucose diet to maintain blood glucose at levels greater than or equal to 30.0 mmol/L and was considered hyperglycaemic. Overall, 5 × 10^6^ T24 cells were implanted in the mice; these mice were monitored every four days. Tumour volumes were estimated using the following formula: Volume = width × length × (width + length)/2. The mice were killed on day 28, and the tumours were dissected out.

In the metastasis experiment, mice were randomly separated into two groups and subcutaneously implanted with 0.1 mL of T24 cells by lateral tail vein (caudal vena) injection. Metastatic progression was monitored weekly. After 28 days, the mice were killed and their lungs were surgically removed.

In the Kaplan‐Meier survival curve experiment, 20 nude mouse models of diabetes were randomly separated into two groups, euglycemic group and a hyperglycaemic group. One nude mouse in the euglycemic group soon died of an infection, which occurred during the establishment process of diabetes model and was excluded. T24 (5 × 10^6^) cells were implanted in the mice. These mice were observed daily to determine mortality. This observation ended 60 days after implantation or when the mice died naturally.

Animal experiments were approved by the Ethics Review Board of The First Affiliated Hospital of Wenzhou Medical University.

### 2.10 Statistical analysis

Statistical analyses were performed using SPSS18.0. The results are shown as means ± the standard error of the mean (SEM). Significance was indicated by *P* < .05.

## 3 RESULTS

### 3.1 Characteristics of BC patients

Table 1 shows the basic characteristics of BC patients based on their blood glucose levels. Among these 200 patients, the mean HbA1c level was 5.2% in the normal glucose tolerance (NGT) group and 8.3% in the T2D group. Fasting glucose was 5.5 nmol/L in the NGT group and 9.1 nmol/L in the T2D group. The data listed were acquired during the patient's first hospitalization due to BC. The average time since first diagnosis of T2D was 14.4 years. Meanwhile, of the 100 BC patients with T2D, 16 patients took metformin (MET), 15 patients took another antidiabetic drug, 49 patients (around half) took more than 2 antidiabetic drugs (including MET), and the remaining 20 patients denied any antidiabetic drug usage.

**TABLE 1:** Characteristics of BC patients

Characteristic	NGT	T2D	*χ* ^2^	*P* value
Age			0.506	.477
≤60	47	42		
>60	53	58		
Gender			0.385	.535
Male	85	88		
Female	15	12		
Histological grade			6.483	.016
G1 + G2	60	42		
G3	40	58		
T staging			14.593	<.001
NMI	62	35		
MI	38	65		
N staging			8.777	.003
N0	92	76		
N1+	8	23		
M staging			2.749	.212
M0	99	95		
M+	1	5		
Tumour size (cm)			0.533	.465
≤3	40	35		
>3	60	65		

Abbreviation: NGT, normal glucose tolerance.

Although the average age and gender were similar in both groups, there were significant differences in some baseline characteristics, including tumour grade and pathological tumour‐node‐metastasis (pTNM) stage. The T2D group showed significantly reduced differentiation (*P* = .016), significantly higher T classification (*P* < .001) and significantly higher N classification (*P* = .003) than the NGT group. No significant differences in M classification and tumour size were found between the two groups (*P* > .05).

### 3.2 YAP1 and TAZ are up‐regulated in BC tissues

**FIGURE 1:**
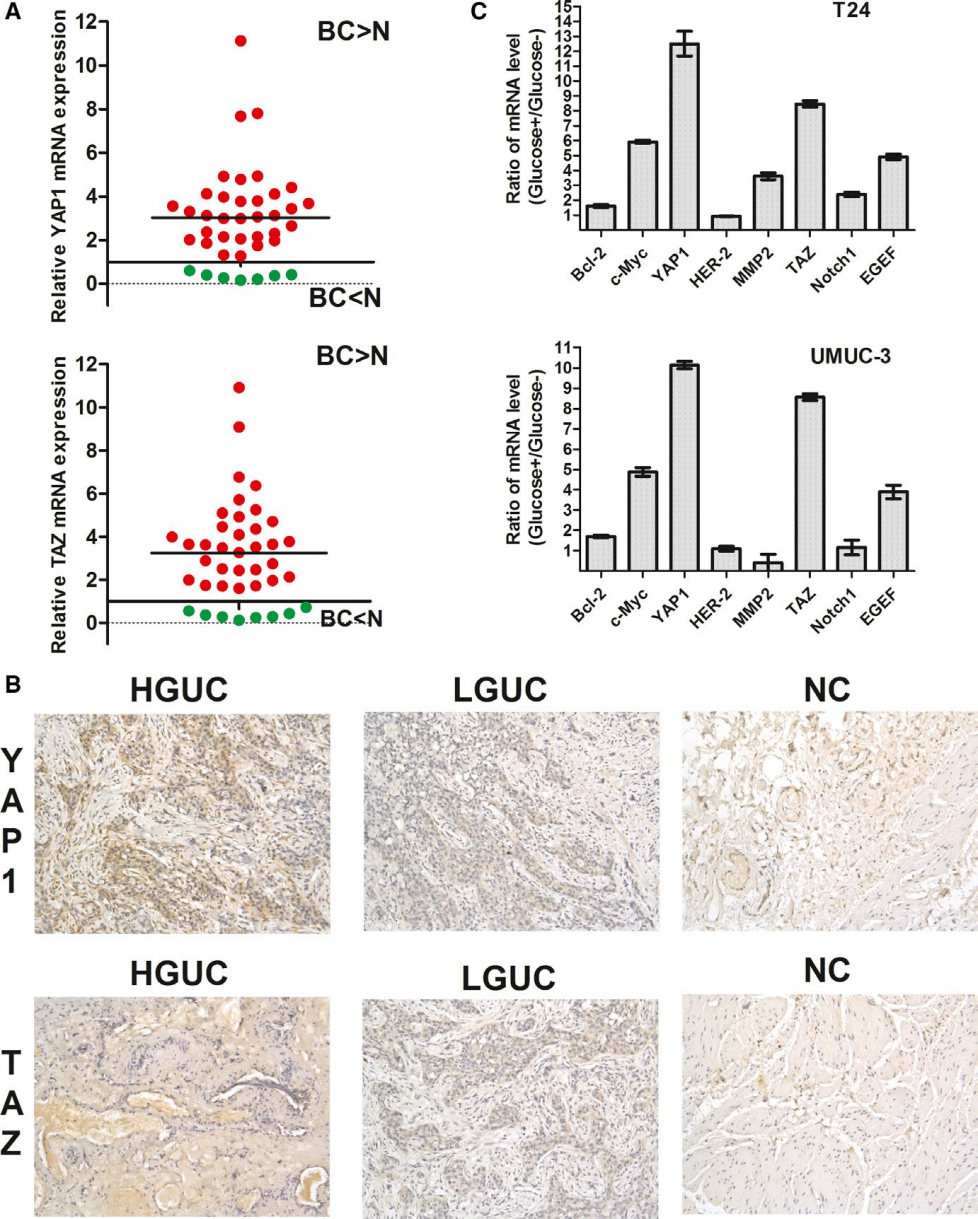
Elevated expression of YAP1 and TAZ in bladder cancer. A, The expression levels of YAP1 and TAZ were higher in tumour tissues than in the surrounding normal tissues based on qPCR. B, YAP1 and TAZ levels were determined in both BC and normal samples using immunohistochemistry (×100). C, The expression of 8 canonical oncogenes was examined using qPCR in T24 and UMUC‐3 cells. After treatment with a high‐glucose concentration solution for 48 h, markedly higher levels of YAP1 mRNA and TAZ mRNA were observed compared with other classical oncogenes. HGUC: high‐grade urothelial carcinoma; LGUC, low‐grade urothelial carcinoma; NC, normal control. *P < .05

qPCR assessment confirmed the significant up‐regulation of YAP1 and TAZ in BC tissues relative to that in adjacent normal tissues (Figure 1A). IHC further confirmed that both YAP1 and TAZ protein expression co‐ordinately increased the pathologic grade of BC (Figure 1B). Furthermore, YAP1 and TAZ expression was higher in BC patients with T2D than in those without (Table 2). qPCR analysis demonstrated that T24 and UMUC‐3 BC cells treated with a high concentration glucose solution for 48 hours exhibited markedly higher YAP1 and TAZ mRNA levels than did other classical oncogenes (Figure 1C).

**TABLE 2:** Expression of YAP1 and TAZ between two groups in BC patients

Protein	Positive cases	Negative cases	*χ* ^2^	*P* value
YAP1			0.003	.001
BC with NGT	77	23		
BC with T2D	93	7		
TAZ			0.004	.002
BC with NGT	72	28		
BC with T2D	89	11		

### 3.3 High‐glucose concentration facilitates EMTs by regulating YAP1 and TAZ

**FIGURE 2:**
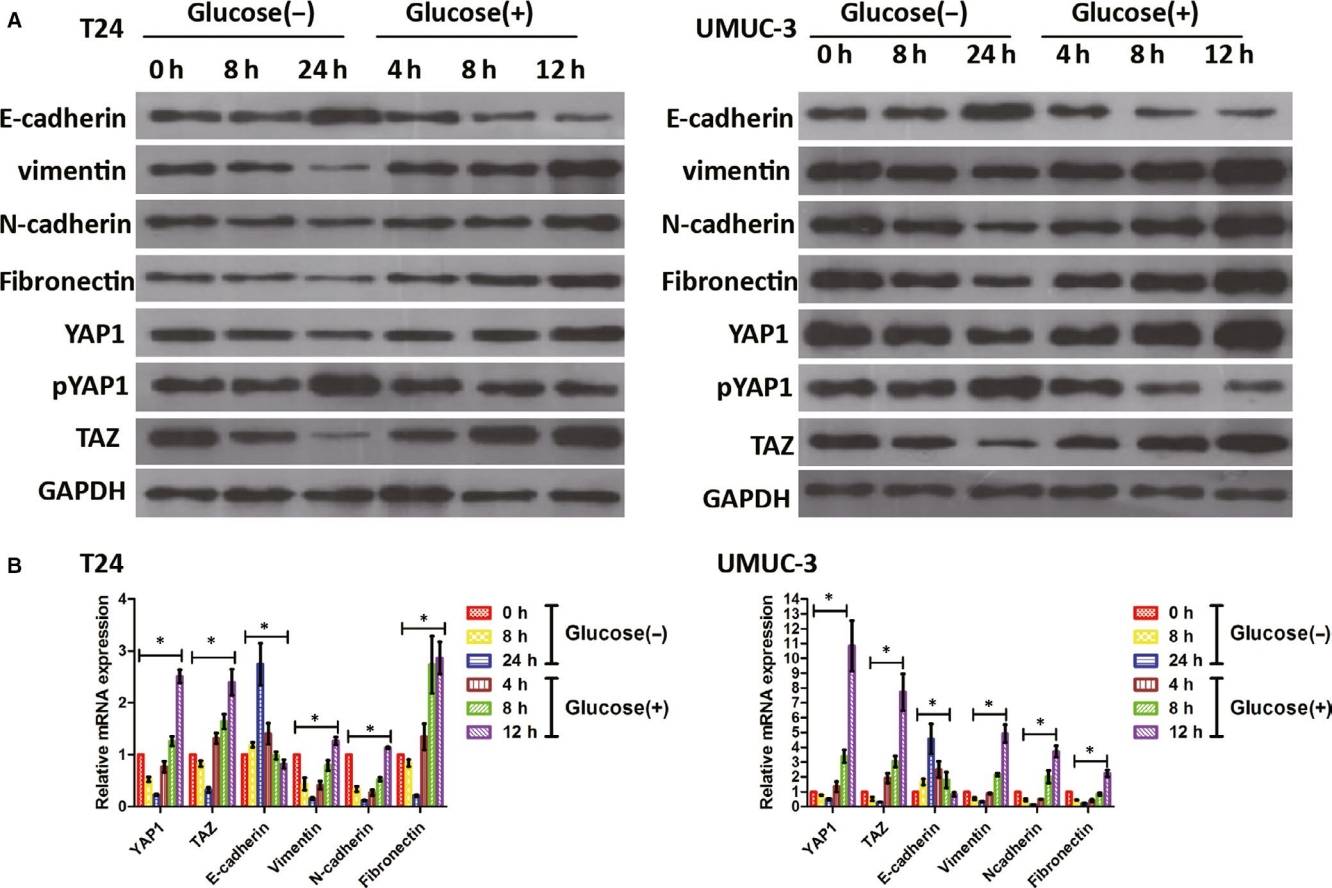
High‐glucose levels regulate EMTs and YAP1/TAZ activity in BC cell lines. T24 and UMUC‐3 cells were starved of glucose (2.8 mmol/L) for the indicated intervals (0, 8, 24 h) and then stimulated with glucose (25 mmol/L) for the indicated intervals (4, 8, 12 h). The expression of YAP1, pYAP1, TAZ, vimentin, N‐cadherin, fibronectin and E‐cadherin was assessed using Western blotting (A) and qPCR (B). *P < .05

To assess the effects of glucose on the functions of YAP1 and TAZ, we used two culture media with differing glucose concentrations: a low glucose concentration of 2.8 mmol/L and a high‐glucose concentration of 25 mmol/L. When BC cells were glucose‐starved (2.8 mmol/L) for periods of 0, 8 and 24 hours, both the protein (Figure 2A) and mRNA (Figure 2B) expressions of YAP1 and TAZ decreased. Simultaneously, the phosphorylation of YAP1 (which produces pYAP1, a depressed state of YAP1 retained in the cytosol^13^) increased in both T24 and UMUC‐3 cells (Figure 2A). After glucose levels were raised (25 mmol/L), YAP1 and TAZ levels increased markedly (Figure 2A,B), while pYAP1 levels decreased (Figure 2A). These results indicate that exposure to high‐glucose levels stimulates YAP1 and TAZ expression and potentially affects YAP1 function.

**FIGURE 3:**
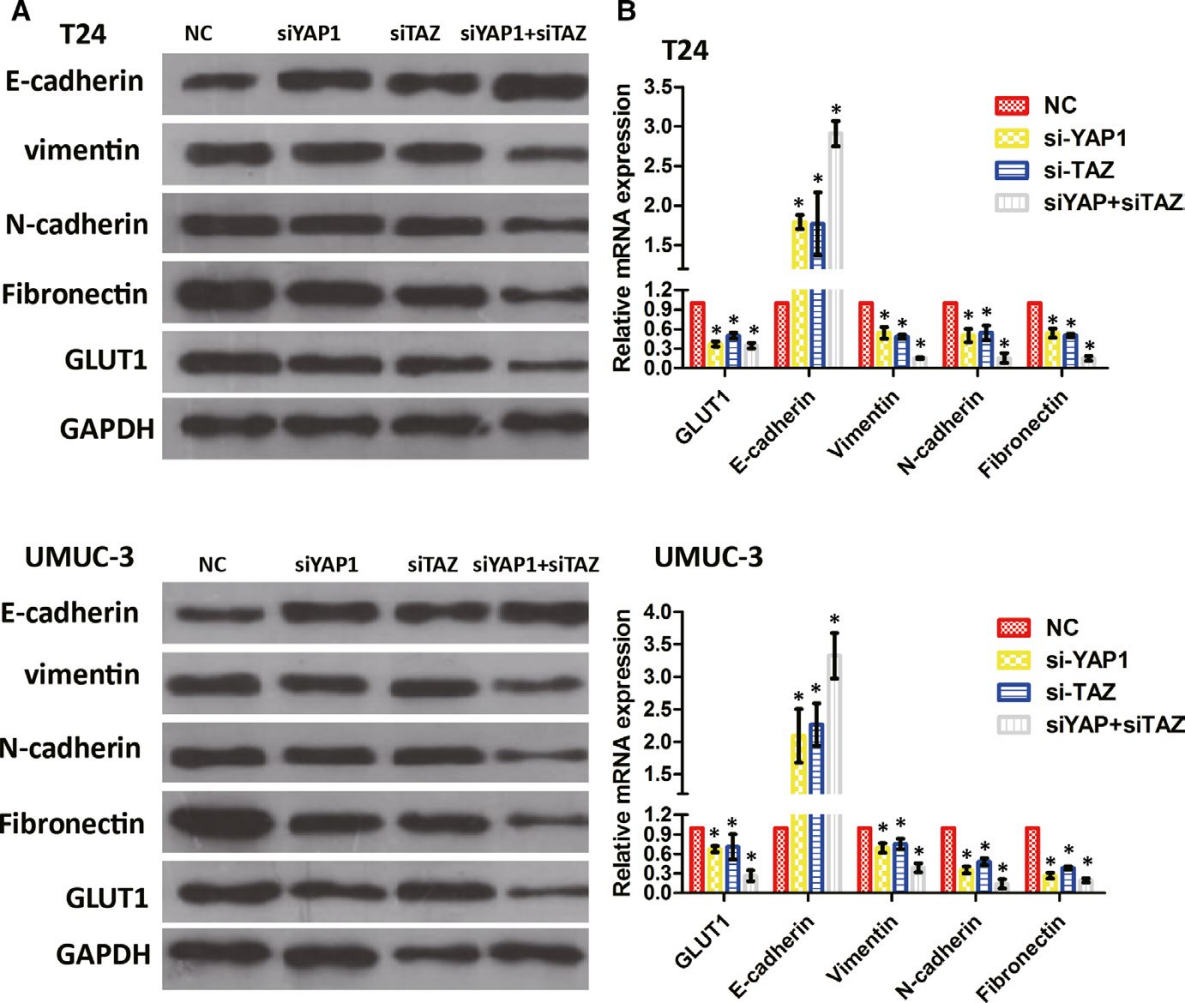
YAP1 and TAZ regulate EMT and GLUT1 in BC cell lines. In T24 and UMUC‐3 cells, decreased GLUT1, vimentin, N‐cadherin and fibronectin expression and increased E‐cadherin expression were observed in siYAP1‐, siTAZ‐ or siYAP1 + siTAZ‐treated cells based on the results of Western blotting (A) and qPCR (B). NC, negative control. *P < .05

The transfection of T24 and UMUC‐3 cells with either siYAP1 or siTAZ altered the expression of the EMT‐associated proteins vimentin, N‐cadherin, fibronectin and E‐cadherin. While the single knockdown of YAP1 and TAZ decreased vimentin, N‐cadherin and fibronectin (and increased E‐cadherin) expression, the most remarkable effect was observed upon cotransfection with both siYAP1 and siTAZ (Figure 3; *P* < .05). These findings demonstrate that YAP1 and TAZ exert noticeable effects on EMT‐associated protein expression.

Furthermore, in addition to altering the expression of YAP1 and TAZ, variable glucose concentrations in the culture media affected the expression of EMT‐related markers in BC cells. Glucose starvation suppressed vimentin, N‐cadherin and fibronectin expression, enhanced E‐cadherin expression, and simultaneously suppressed YAP1 and TAZ expression. In addition to the restoration of YAP1 and TAZ levels, the re‐addition of glucose promoted EMTs, as evidenced by increased vimentin, N‐cadherin and fibronectin, as well as decreased E‐cadherin expression detected via Western blotting (Figure 2A) and qPCR (Figure 2B; *P* < .05). Together, these results suggest that high‐glucose levels promote EMTs by regulating YAP1 and TAZ.

### 3.4 YAP1 and TAZ regulate GLUT1 in high‐glucose culture media

To investigate the role of YAP1 and TAZ in glucose metabolism, YAP1, TAZ or both (YAP1 and TAZ) were knocked down in T24 and UMUC‐3 cells via RNA Silencing. GLUT1 expression was obviously decreased upon YAP1 or TAZ inhibition (Figure 3A,B), with an even more obvious decrease observed in cells cotransfected with siYAP1 and siTAZ (*P* < .05). These findings indicate that YAP1 and TAZ may be involved in glucose metabolism via GLUT1 regulation.

### 3.5 MET impairs high‐glucose‐induced EMTs in BC cells

**FIGURE 4:**
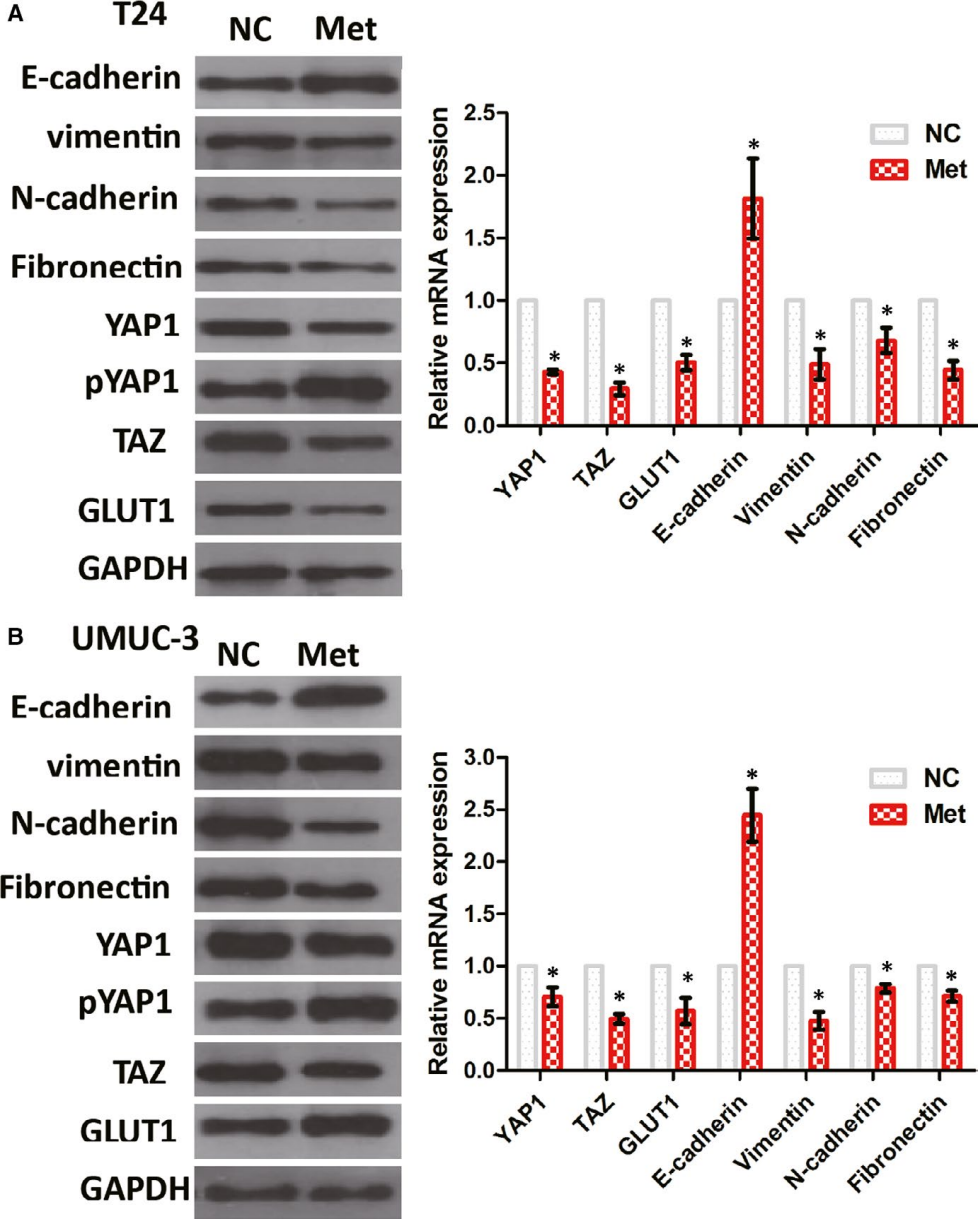
MET impaired high‐glucose‐induced EMTs in BC cells. In T24 (A) and UMUC‐3 (B) cell lines, MET at least partially reduced EMTs and the expression of YAP1, TAZ and GLUT1 induced by high‐glucose levels. The expression of YAP1, pYAP1, TAZ, E‐cadherin, vimentin, N‐cadherin, fibronectin and GLUT1 was assessed using Western blotting and qPCR. *P < .05

Our results showed that high‐glucose levels altered EMT‐associated protein expression via YAP1/TAZ, and consequently potentially promote EMTs, while treatment with MET inhibited EMTs in UMUC‐3 and T24 cells. Both cell lines displayed decreased vimentin, N‐cadherin, fibronectin, YAP1, TAZ and GLUT1 expression and increased E‐cadherin expression under high‐glucose conditions following MET treatment (Figure 4A,B; *P* < .05).

### 3.6 High‐glucose levels promote the invasion and proliferation of BC cell lines

**FIGURE 5:**
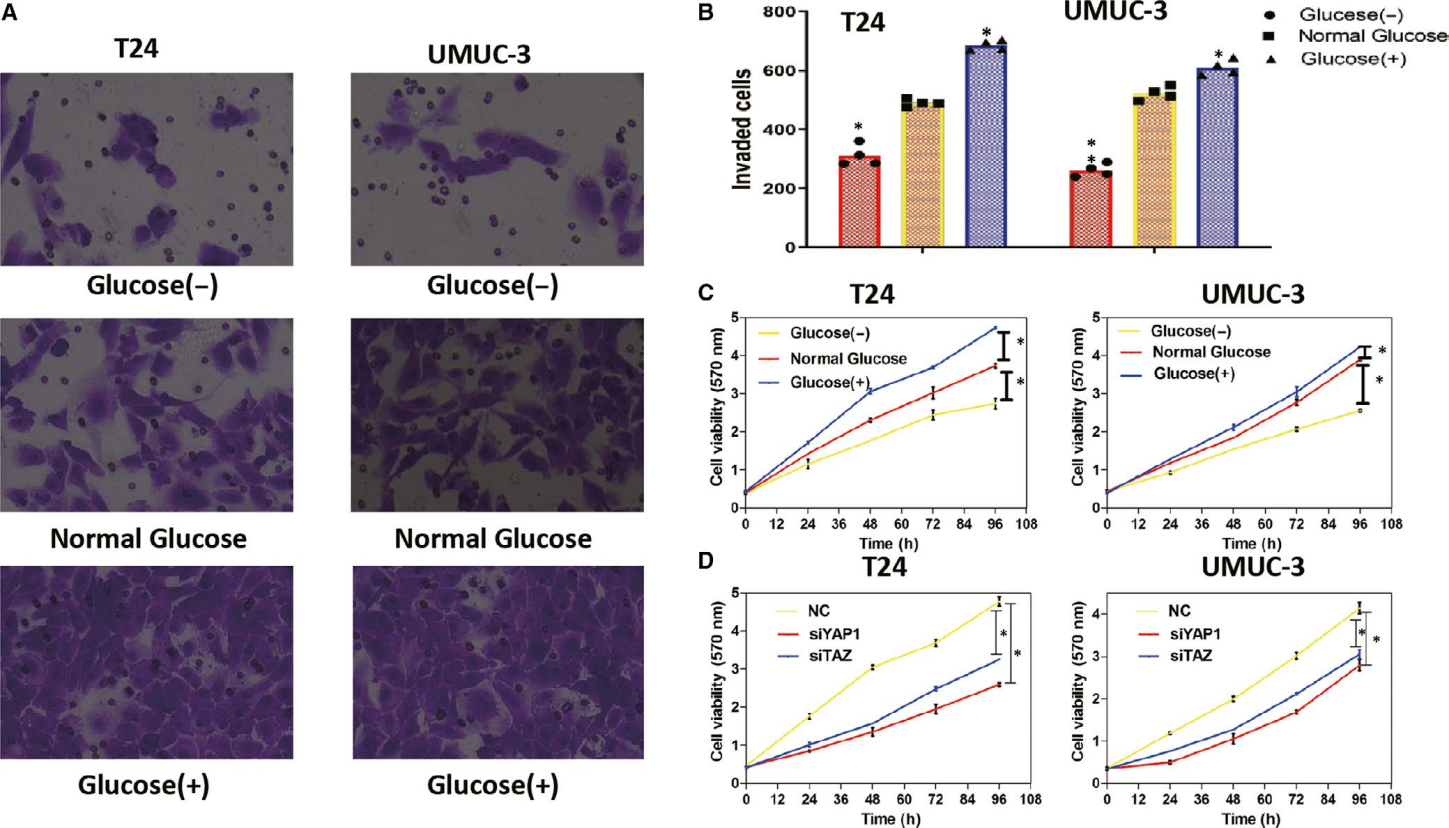
High glucose promotes the invasion and proliferation of BC cell lines. A and B, The invasive ability of T24 and UMUC‐3 cells was inhibited by low glucose levels and was enhanced by high‐glucose levels based on Transwell assays. C, T24 and UMUC‐3 cell viability was inhibited by low glucose levels and enhanced by high‐glucose levels based on MTT assays. D, In high‐glucose culture media, cell viability decreased after siYAP1 or siTAZ treatment. *P < .05

The number of UMUC‐3 and T24 cells that invaded through the Matrigel was determined using Transwell assays under low (2.8 mmol/L), normal (11.2 mmol/L) and high‐glucose (25 mmol/L) conditions. For both cell lines, cells cultured in high‐glucose media were significantly more invasive and cells grown in low glucose media were significantly less invasive than cells cultured in normal glucose media (Figure 5A,B; *P* < .05).

Similarly, both cell lines exhibited enhanced proliferation under high‐glucose conditions and reduced proliferation under low glucose media conditions based on MTT assays (Figure 5C; *P* < .05). Furthermore, YAP1 or TAZ knockdown suppressed the proliferation of both BC cell lines, even under high‐glucose conditions (Figure 5D; *P* < .05).

### 3.7 Hyperglycaemia promotes tumour growth and metastasis in vivo

**FIGURE 6:**
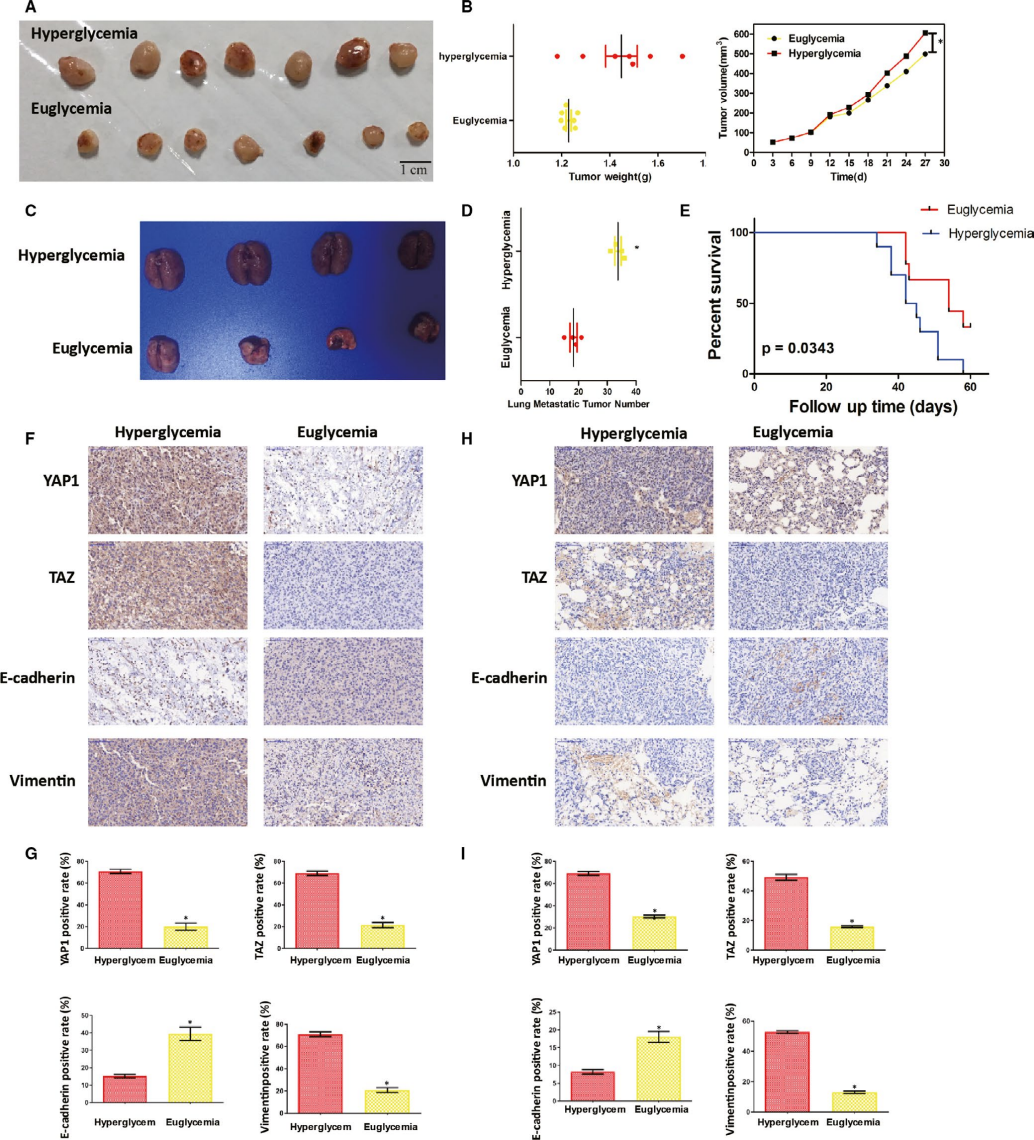
High‐glucose levels drive tumour growth and metastasis in vivo. A, STZ‐induced xenograft nude mouse models of diabetes were established. T24 BC cells were subcutaneously injected into the back of mice to simulate carcinogenesis. The volumes of the xenotransplanted tumours in the two groups were compared at 28 d post‐implantation (n = 7). B, Tumour weights and volumes were measured in the two groups over a period of 28 d. C and D, T24 cells were injected into the tail veins of mice (5 × 10^6^ cells, 4 mice per group). After 28 d, the mice were killed and the lungs were removed and fixed in picric acid prior to being photographed. The number of lung metastatic tumours was then counted. E, Kaplan‐Meier survival curves for the mice in the two different blood glucose level groups. F, and G, Representative image of the IHC staining of xenotransplanted tumours and protein staining scores for the two groups. H and I, Representative image of the IHC staining of lung metastases in the two groups and protein staining scores in each group. *P < .05

In our STZ‐induced xenograft nude mouse model of diabetes, we observed that the weight and volume of tumours were markedly higher in the hyperglycaemic mice than in the euglycemic mice (Figure 6A,B; *P* < .05). Histological analysis revealed that the expression of YAP1, TAZ and vimentin was higher and the expression of E‐cadherin was reduced in the tumour tissues of hyperglycaemic mice (Figure 6F,G; *P* < .05).

The metastasis experiment was performed two months after T24 cell injection, and post‐mortem examination indicated that hyperglycaemia significantly promoted BC metastasis in vivo. The number of lung metastases was much greater in the hyperglycaemic group (Figure 6C,D; *P* < .05); histological examination suggested pulmonary metastasis. A similar result was observed in metastatic tumours as well as in subcutaneous transplanted tumours (Figure 6H,I, *P* < .05). Furthermore, hyperglycaemic mice had reduced lifespans relative to euglycemic mice (Figure 6E).

Collectively, these results indicate that hyperglycaemia promoted both BC cell proliferation and the development of metastases and reduced life span in vivo.

## 4 DISCUSSION

Although the treatment of BC has improved over time, including the development of new surgical techniques and targeted drugs, due to the high rates of recurrence and distant metastasis associated with the disease, further advances are still necessary. Therefore, deeper understanding of the molecular mechanisms underlying BC and the identification of novel pathological targets are essential to improving BC treatment.

Glucose is the most important nutrient for cellular energy. Due to the high‐glucose consumption of cancer cells, cancer progression is positively associated with impaired energy metabolism.^19^ T2D is characterized by high blood glucose levels and is positively correlated with the initiation, promotion and progression of various human cancers.^7^, ^8^ High‐glucose levels can impair angiotensinogen and enhance breast cancer proliferation and metastasis.^20^ Conversely, glucose restriction can inhibit various energy‐dependent pathways such as IGF‐1/PI3K/Akt/mTOR in cancer cells,^21^ suppress cell metabolism and growth, and promote G1 phase arrest and apoptosis.^22^ Overall, several studies have correlated high‐glucose levels/T2D with cancer progression. Our study demonstrates that pathologic grade and TNM stage were higher in BC patients with T2D than in BC patients without T2D. Additionally, high‐glucose levels significantly enhanced both BC cell proliferation and invasion in vitro and BC tumour growth and metastasis in vivo. A previous pooled analysis of data from 22 randomized controlled trials (RCTs) suggested that newly diagnosed cases of BC occur in a greater proportion of patients treated with dapagliflozin compared to the incidence in patients receiving placebo or a comparative treatment.^23^ However, this conclusion was weak and controversial^24^ and most studies have demonstrated that T2D itself, rather than antidiabetic drugs, is a risk factor for cancer. Metastasis and proliferation play essential roles in cancer development and are considered hallmarks of the condition.^25^ In BC, distant metastasis generally precedes an extremely poor prognosis.^26^ EMT, a cellular programme crucial for embryogenesis and wound healing, is a pivotal process in cancer metastasis.^25^ High‐glucose levels have also been confirmed to promote EMTs in a differing set of benign diseases.^9^, ^10^ This may be partial because high‐glucose levels and dysregulated glucose metabolism result in an acidic microenvironment, which promotes extracellular matrix degradation and impairs cell adhesion, consequently facilitating EMTs.^27^ Because of the substantial glucose consumption of cancer cells, some studies have focused on the positive effects of high‐glucose levels in cancer development.^28^, ^29^ Our study demonstrates that high‐glucose levels up‐regulated vimentin, N‐cadherin and fibronectin expression, and down‐regulated E‐cadherin expression. As a result, cell proliferation and invasion in vitro, and tumour growth and metastasis in vivo were notably increased by high‐glucose levels (hyperglycaemia). As a standard antidiabetic drug, MET partially suppressed the pro‐EMT effects of high‐glucose levels. Together, these results suggest that the pro‐EMT effects induced by high‐glucose levels may contribute to BC progression, while targeting glucose metabolism with molecular interventions may be a viable strategy for BC therapy, particularly in BC patients with T2D. Furthermore, among BC patients with T2D, tight glucose control may reduce metastasis and improve outcomes.

The Hippo signalling pathway is a canonical pathway that regulates cellular proliferation, differentiation and apoptosis and plays a crucial role in carcinogenesis.^30^ YAP1 and TAZ, the key components of this signalling pathway, are associated with tumour development in various human cancers.^31^ Previous studies have indicated that YAP1 and TAZ play important roles in cancer cell progression by influencing EMT. In breast cancer, YAP1 can directly interact with ZEB1, (a crucial stimulator of EMT processes) and thereby increases metastatic risk.^15^ In osteosarcoma, a positive feedback loop of EMT regulation by TAZ and miR‐135b has been confirmed.^16^ Our study demonstrated that YAP1 and TAZ levels were positively correlated with pathologic grade in BC. In BC cell lines, EMT‐associated protein levels were altered after the knockdown of YAP1 or TAZ. Previous studies on YAP1/TAZ and glucose metabolism have mostly focused on T2D complications and T2D itself. Multiple studies have confirmed the role of YAP1 and TAZ (in addition to the pancreas and pancreatic β cells) in glucose metabolism.^32^ In human embryonic kidney cells (HEK293A), glucose starvation could inhibit YAP transcriptional activity and YAP‐dependent transformation via AMP‐activated protein kinase (AMPK) regulation.^33^ However, little is known about the interaction between high‐glucose levels and the Hippo pathway in cancer progression, especially in BC. In breast cancer, YAP1 and TAZ activity increases when cells actively incorporate glucose.^34^ We confirmed that high‐glucose levels promote EMT by increasing YAP1 and TAZ activity and that MET could partially suppress this effect. As a first‐line pharmacotherapy for T2D, previous studies have confirmed that MET also inhibits cancer progression. This anticancer effect has been associated with its antidiabetic function and the mTOR pathway involved in its progression.^35^, ^36^ In addition to high‐glucose levels affecting YAP1 and TAZ expression, the Hippo pathway also regulates glucose metabolism.^32^ In our study, GLUT1 (a transporter responsible for glucose uptake in cells) levels were found to be positively associated with YAP1 and TAZ expression.

**FIGURE 7:**
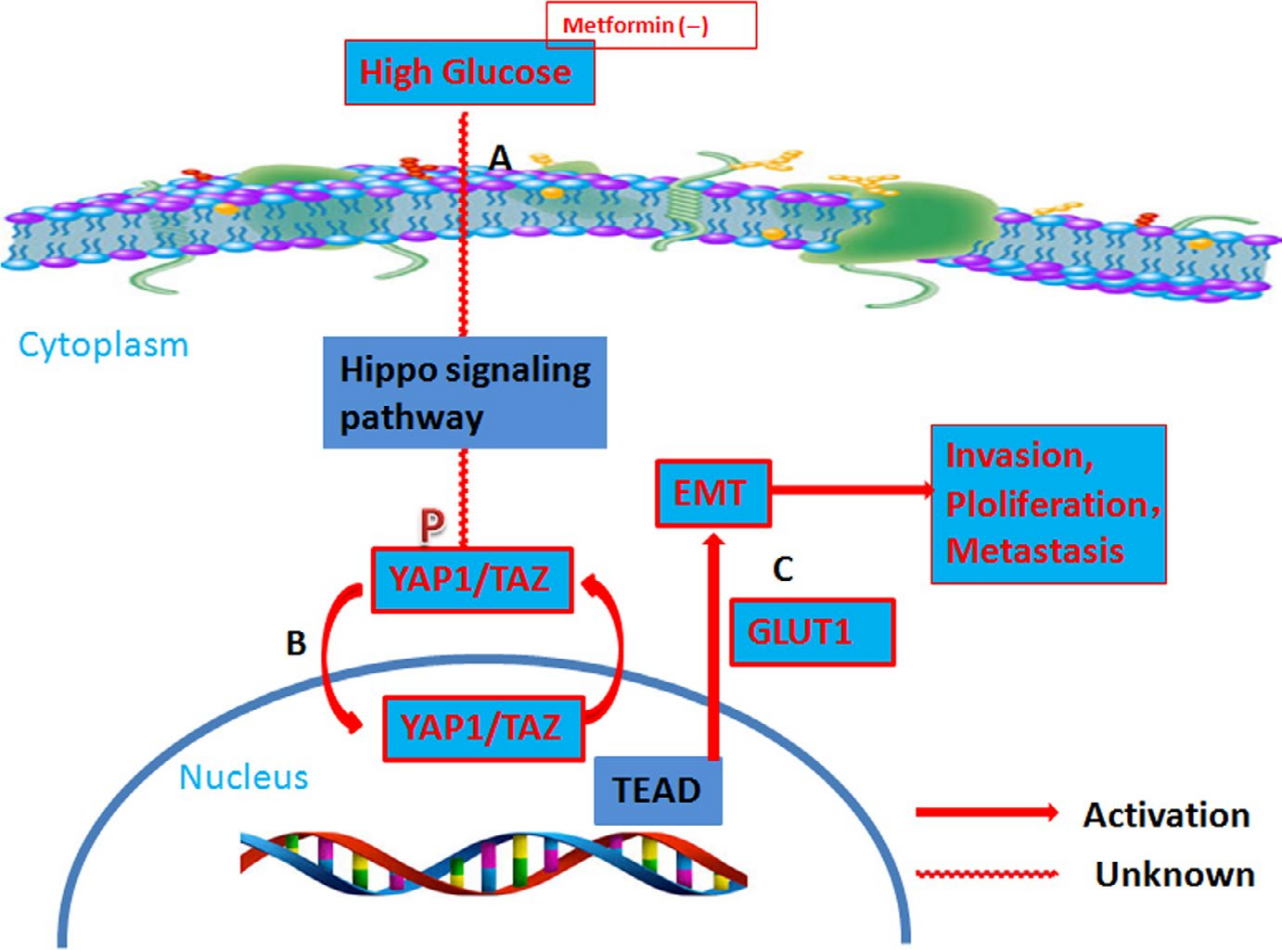
Glucose promotes EMTs in BC by regulating the functions of YAP1 and TAZ. A, High‐glucose levels promoted the activity of the Hippo signalling pathway in BC cells, an effect inhibited by MET. B, Under high‐glucose conditions, YAP1 and TAZ levels increased markedly, while pYAP1 levels decreased. This suggests that large amounts of YAP1 and TAZ are translocated into the nucleus where they enhance TEAD‐mediated transcription and perform their functions. C, EMTs were more common and GLUT1 levels were up‐regulated in BC cell lines, facilitating their invasion and proliferation

In summary, our results indicate that T2D is positively correlated with the pathologic grade and pTNM stage of BCs. High‐glucose levels promote cell proliferation and invasion in vitro, and tumour cell proliferation and metastasis in vivo. High‐glucose levels increased vimentin, N‐cadherin and fibronectin expression, and decreased E‐cadherin expression through YAP1 and TAZ regulation, promoting EMTs in BC cells. MET partially suppressed the pro‐EMT effects of high‐glucose levels. Finally, YAP1 and TAZ were found to regulate GLUT1 expression (Figure 7). Therefore, the Hippo signalling pathway may act as a switch that controls the carcinogenic effect of dysfunctional glucose metabolism in BC. Thus, targeting this pathway could preferentially be exploited as a therapeutic strategy for BC.

## 5 CONCLUSIONS

In this study, we investigated the function and effects of high‐glucose levels on EMTs in BC. Our study demonstrated that YAP1 and TAZ are key targets of high‐glucose‐induced EMTs in BC. These novel findings suggest that diabetes management could have a significant impact in cases of BC and T2D comorbidity and that targeting the key effector molecules of high‐glucose‐induced EMTs may be of therapeutic value in these patients.

## CONFLICT OF INTEREST

The authors declare that they have no competing interests.

## AUTHOR CONTRIBUTION

**Shi Li:** Conceptualization (lead); Funding acquisition (equal); Writing‐original draft (lead); Writing‐review & editing (equal). **Hua Zhu:** Data curation (equal); Methodology (equal). **Hongde Chen:** Formal analysis (equal); Project administration (equal); Resources (equal). **Jianling Xia:** Funding acquisition (equal); Methodology (equal); Project administration (equal); Resources (equal); Writing‐review & editing (equal). **Fangyi Zhang:** Data curation (equal); Formal analysis (equal); Investigation (equal). **Ruoting Xu:** Formal analysis (equal); Methodology (equal). **Qi Lin:** Formal analysis (equal); Methodology (equal).

## ETHICAL APPROVAL

This study was reviewed and approved by the Ethical Board at the First Affiliated Hospital of Wenzhou Medical University and the Ethical Board at the Sichuan Provincial People's Hospital.

## Supporting information

Supplementary Material

## Data Availability

All data generated or analysed during this study are included in this published article.
