# Supplementary material for: Glucose promotes epithelial‐mesenchymal transitions in bladder cancer by regulating the functions of YAP1 and TAZ
Source: J Cell Mol Med. 2020 Jul 17;24(18):10391–401. doi: 10.1111/jcmm.15653 (PMC7521329; doi:10.1111/jcmm.15653)
Supplement: Supplementary file 1 — Supplementary Material [file JCMM-24-10391-s001.docx]

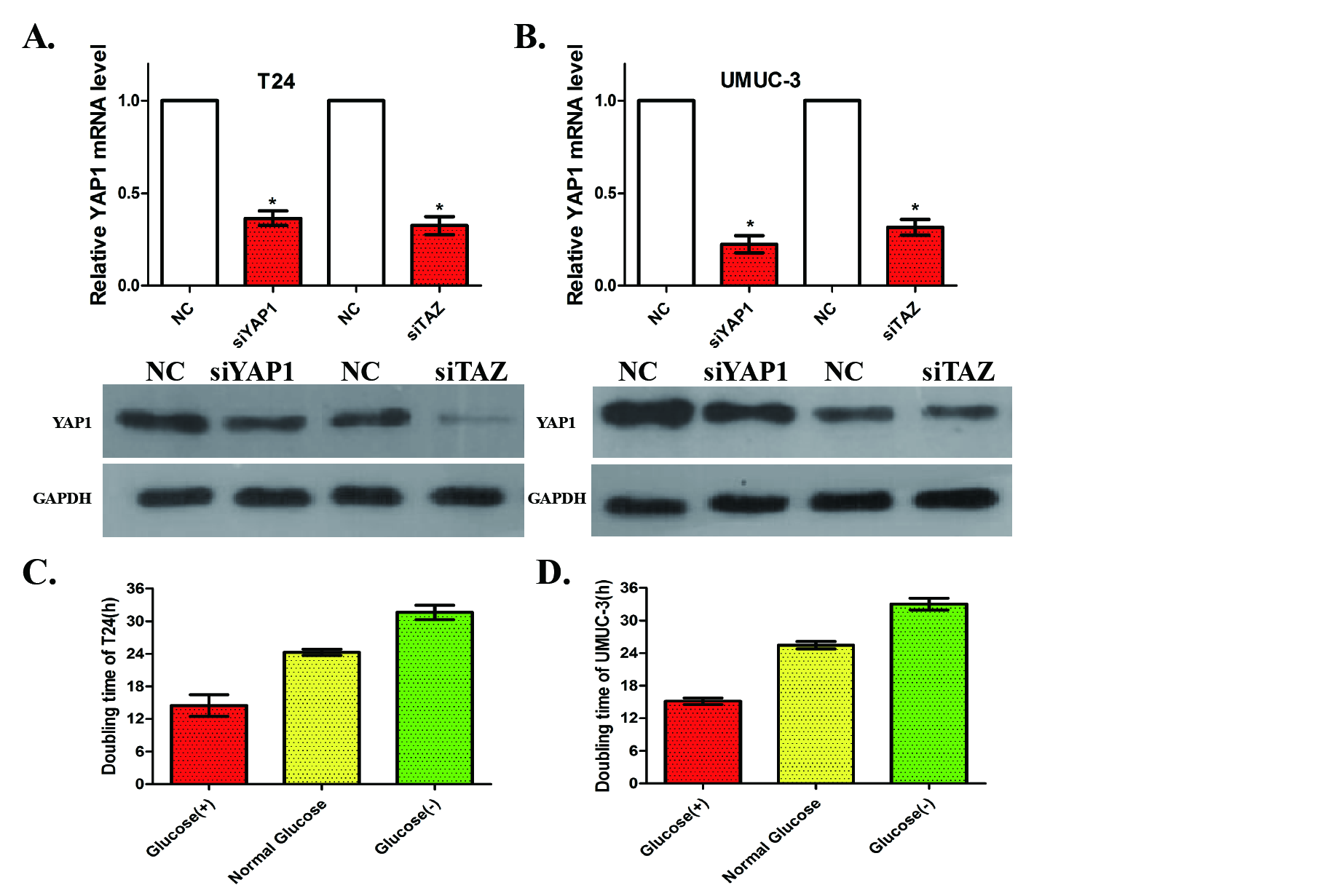


**FIGURE S1** YAP1 or TAZ expression decreased in siYAP1 or siTAZ treated bladder cancer cells, respectively, (A:T24 and B:UMUC-3) as measured by qPCR and western blotting. The doubling time of T24 (C) and UMUC-3 (D) in culture media with different glucose concentrations. NC: negative control, * = p<0.05


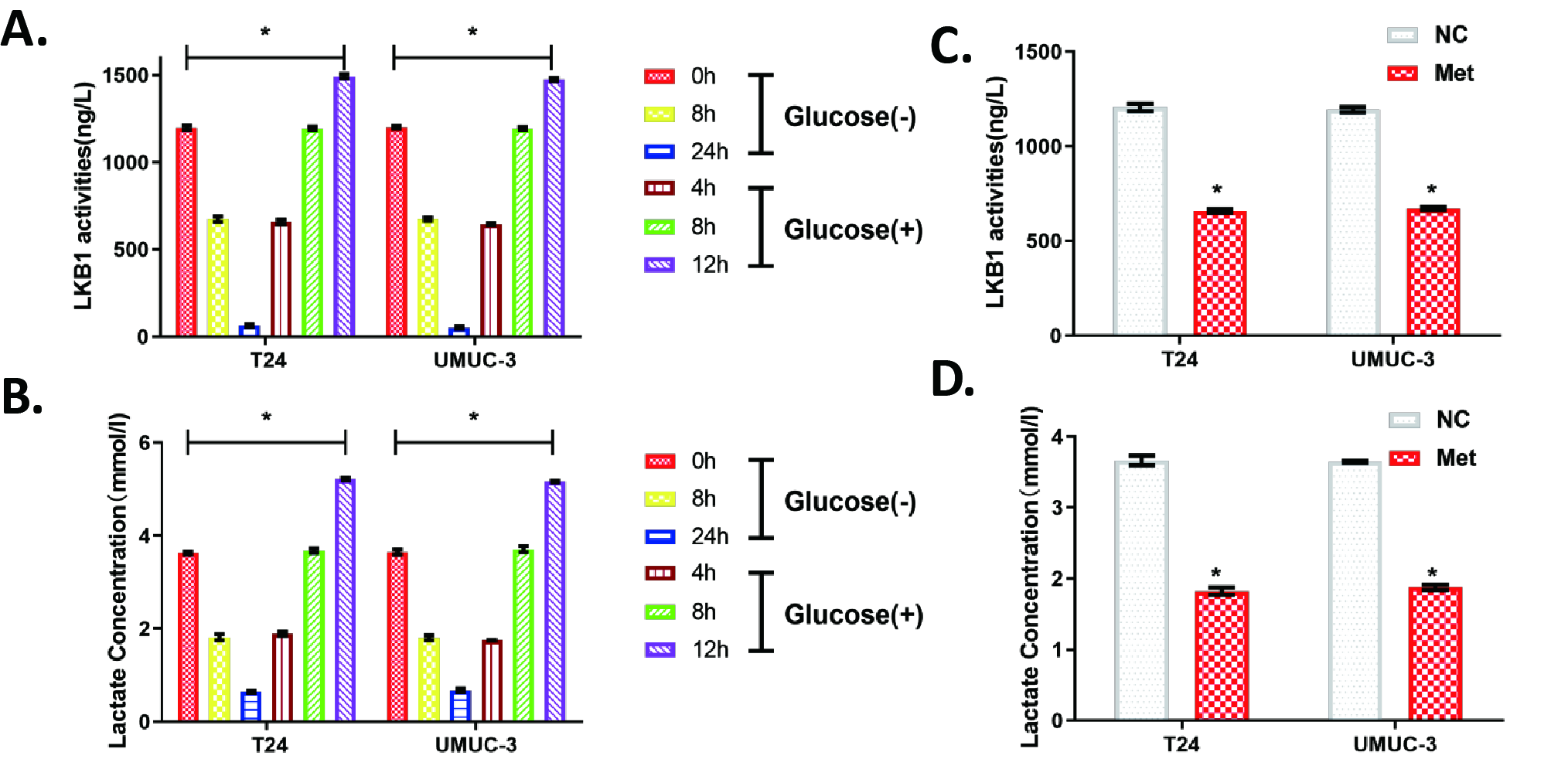


**FIGURE S2** LKB1 activities and lactate concentration in BC cell lines.

T24 and UMUC-3 cells were starved of glucose (2.8 mM) for the indicated intervals (0, 8, 24 h) and then stimulated with glucose (25 mM) for the indicated intervals (4, 8, 12 h). The LKB1 activities (A) and lactate concentration (B) were assessed at different time points. The changes of LKB1 activities (C) and lactate concentration (D) were detected after MET treatment. NC: negative control,* = p<0.05

**TABLE S1** Primer sequences for real-time PCR

| Gene name | Primer sequences (5' to 3') | Primer length (bps) |
| --- | --- | --- |
| YAP1 | F- CGCTCTTCAACGCCGTCA  R- AGTACTGGCCTGTCGGGAGT | 18  20 |
| TAZ | F-GTCACCAACAGTAGCTCAGATC  R- GATTACAGCCAGGTTAGAAAG | 22  21 |
| E-cadherin | F- TGTCCGCCCCGACTTGTCTCTC  R- GTCCTCTGGCCCCAGCCTCTCT | 22  22 |
| Vimentin | F- GTGGACCAGCTAACCAACGACAAA  R- TTCAAGGTCAAGACGTGCCAGAGA | 24  24 |
| N-Cadherin  Fibronectin  GLUT1  GAPDH | F-TGGGAAATGGAAACTTGATGGC  R-TGGAAAGCTTCTCACGGCAT  F- ATGATGAGGTGCACGTGTGT  R- CCCTGACCGAAGCATGTACA  F- GGCTTCTCCAACTGGACCTC  R-CCGGAAGCGATCTCATCGAA  F-GACTCATGACCACAGTCCATGC  R-AGAGGCAGGGATGATGTTCTG | 22  20  20  20  20  20  22  21 |
